# THE PROMISE AND CHALLENGES OF DIGITAL INNOVATIONS TO SUPPORT OLDER ADULTS AND CAREGIVERS

**DOI:** 10.1093/geroni/igae098.1841

**Published:** 2024-12-31

**Authors:** Laura Gitlin, Walter Boot

**Affiliations:** Chair; Discussant

## Abstract

Online platforms are promising scalable approaches for delivering interventions addressing critical public health needs. As most older adults and caregivers utilize smartphones, tablets or computers, this approach can improve access to on-demand support. However, there are numerous challenges for advancing digital innovations. This symposium showcases five digital platforms that target different populations (caregivers, socially isolated, individuals with emotional needs); address different needs (monitoring, support, care strategies); have different developmental approaches, and are in different testing phases (feasibility, usability, acceptability, efficacy). Ms. Webster first presents national recruitment efforts to enroll 262 caregivers to evaluate an online platform, WeCareAdvisor, that provides strategies to manage dementia-related behavioral symptoms. She illustrates the volume needed for enrollment and biases of recruitment approaches. Next, Dr. Czaja discusses processes for developing PRISM, an effective online program addressing social isolation. Highlighted is the importance of end-user engagement throughout development and testing. Dr. Sadarangani presents CareMobi, which enables families using adult day services to track their relatives care in that setting. She highlights the feasibility of staff and families to use the app to improve communications. Dr. Jain next presents on adapting late-life psychotherapies for delivery via mobile devices. His study demonstrates the effectiveness of adapting approaches for delivery in this modality and opportunities for symptom monitoring. Finally, Dr. Stevens, presents utilization patterns of his online platform, GamePlan4Care, which adapts the NIH REACH caregiver intervention for delivery online. Our nationally recognized expert on technology and aging, Dr. Walter Boot, will discuss the promises and challenges of digital solutions. Behavioral Interventions for Older Adults Interest Group Sponsored Symposium

